# Impact of Annual Wellness Visits on Preventing Falls and Fractures in Older Adults with Alzheimer's Disease and Related Dementias

**DOI:** 10.1002/alz70858_097560

**Published:** 2025-12-24

**Authors:** Mukaila A Raji, Sheheryar Ali, Yong Shan, Ming Tzeng, Yong‐Fang Kuo

**Affiliations:** ^1^ University of Texas Medical Branch, Galveston, TX, USA

## Abstract

**Background:**

Falls and fractures are leading causes of disability and premature death among older adults with Alzheimer's Disease and Related Dementias (ADRD). One approach to screen for and manage falls/fracture‐related risk factors is the Annual Wellness Visits (AWVs)—which Medicare started reimbursing in 2011. This study evaluates associations between AWV and prevention of falls and fractures among Medicare beneficiaries with ADRD.

**Method:**

We analyzed a cohort of Medicare beneficiaries in 2017 aged ≥68 years with ADRD and continuous enrollment from 2014 to 2016 (*n* = 1,610,637). AWV recipients were stratified by the number of visits in the three years before 2017 (0, 1, 2, or ≥3). Kaplan‐Meier methods estimated rates of falls and fractures. Patients were censored at the end of study (12/31/2021), if they lost Medicare coverage, or switched to HMO, whichever happened first. We used inverse probability treatment weighting (IPTW) with propensity score in Cox proportional hazards models to assess the effect of AWVs on the outcomes.

**Result:**

Numbers of AWVs were associated with reduced risks for falls among ADRD beneficiaries (HR: 0.990 for 1 AWV; 0.975 for 2 AWVs; 0.963 for ≥3 AWVs) and fractures (HR: 0.969 for 1 AWV; 0.966 for 2 AWVs; 0.959 for ≥3 AWVs). Increasing AWV frequency was associated with an increase in magnitude of fall risk reduction such that ADRD beneficiaries with ≥ 3 AWVs had a 3.7% reduction in fall risk (vs 1% risk‐reduction with 1 AWV). However, the reduction in fracture were similar, regardless of numbers of AWVs. Analyzing AWV as a time‐dependent covariate in the follow‐up period showed a much larger effect of AWV on magnitude of fall and fracture risk reduction (HR: 0.921 and 0.929—respectively).

**Conclusion:**

Our studies indicate AWVs are associated with lower risks of falls and fractures in older adults with ADRD. The higher the number of AWVs in the three years before 2017, the higher the magnitude of reduction in fall risk. Continuously receiving AWV in the follow‐up period further reduced the risk of fall and fracture. This study emphasizes the need to expand public awareness of AWVs potential impact on fall/fracture prevention in older adults with ADRD.

